# Electrospun Cyclodextrin/Poly(L-lactic acid) Nanofibers for Efficient Air Filter: Their PM and VOC Removal Efficiency and Triboelectric Outputs

**DOI:** 10.3390/polym15030722

**Published:** 2023-01-31

**Authors:** Sompit Wanwong, Weradesh Sangkhun, Pimsumon Jiamboonsri

**Affiliations:** 1 Materials Technology Program, School of Energy, Environment and Materials, King Mongkut’s University of Technology Thonburi, 126 Pracha Uthit Road, Bang Mod, Thung Khru, Bangkok 10140, Thailand; 2 Faculty of Medicine, King Mongkut’s Institute of Technology, Bangkok 10520, Thailand

**Keywords:** poly(L-lactic acid), cyclodextrin, electrospinning, PM, VOC, triboelectric performance, nanogenerator, eco-friendly, air filter

## Abstract

In this work, PLLA and CD/PLLA nanofibers were fabricated using electrospinning and utilized as a particulate matter (PM) and volatile organic compounds (VOCs) filter. The electrospun PLLA and CD/PLLA were characterized with various techniques, including SEM, BET, FTIR, XRD, XPS, WCA, DSC, tensile strength testing, PM and VOCs removal efficiency, and triboelectric performance. The results demonstrated that the best air filter was 2.5 wt%CD/PLLA, which performed the highest filtration efficiencies of 96.84 ± 1.51% and 99.38 ± 0.43% for capturing PM_2.5_ and PM_10_, respectively. Its PM_2.5_ removal efficiency was 16% higher than that of pure PLLA, which were contributed by their higher surface area and porosity. These 2.5 wt%CD/PLLA nanofibers also exhibited the highest and the fastest VOC entrapment. For triboelectric outputs, the 2.5 wt%CD/PLLA-based triboelectric nanogenerator provided the highest electrical outputs as 245 V and 84.70 μA. These give rise to a three-fold enhancement of electrical outputs. These results indicated that the 2.5 wt%CD/PLLA can improve surface charge density that could capture more PM via electrostatic interaction under surrounding vibration. Therefore, this study suggested that 2.5 wt%CD/PLLA is a good candidate for a multifunction nanofibrous air filter that offers efficient PM and VOC removal.

## 1. Introduction

Nowadays, air pollution, including PM and VOCs, and the coronavirus disease 19 (COVID-19) outbreak have become a threatening problem to public health. The World Health Organization (WHO) estimated 7 million people die per year from outdoor and indoor air pollution [1]. In particular, the micro-size of PM with an aerodynamic diameter of less than 2.5 µm (PM_2.5_), which can penetrate deeply into alveoli, has been associated with several diseases, such as cancer [2], respiratory illnesses [3,4], and cardiovascular diseases [5]. To protect vulnerable people from this harmful situation, the demand and usage of face masks and air filters significantly increased worldwide. The current commercial face masks, which were made of polypropylene (PP), non-woven fabric as a filter layer, are designed to be single-use. Consequently, disposable PP masks have become a huge global waste because they are slowly decomposed and converted into microplastic that remains in environments for over 450 years [6]. Another example commercial air filter is high-efficiency particulate air (HEPA) filter, which is the most effective medium to capture particles on a fibrous or porous membrane in an air purifier machine. However, the common filtration media, which is made of fibrous borosilicate glass filter paper, is fragile and has water damage [7]. In addition, both commercial PP and HEPA filters contain fibers in micron size; thus, they typically have a high thickness, leading to a high-pressure drop. In recent years, nanofibers have become attractive materials for air filtration [8]. The nanofibrous air filter offers several advantages, including high surface area, low-pressure drop, low thickness, and lightweight. Based on the filtration theory of fibrous air filters, there are five mechanisms for particle capture, including diffusion, interception, impaction, gravitational settling, and electrostatic attraction [9,10,11]. Generally, filtration mechanisms mainly depend on the structure of the air filter to physically remove PM by interfering with the air stream, but the electrostatic attraction mechanism offers an advantage in improving the filtration performance of filter media by pulling charged particulates from the air to the filter media [9,10,11]. In addition, recent works reported that nanofiber air filters that have triboelectric effect or piezoelectric effect can efficiently enhance PM filtration efficiency [11,12,13]. To manufacture nanofibrous air filters, electrospinning is recognized as a simple and versatile method to fabricate the nanofiber. For biodegradable and compostable polymers, electrospun of various bio-based polymers, such as cellulose, chitosan, silk, and poly (lactic acid), have been reported for PM filtration application [13,14,15]. Among these, poly(L-lactic acid) (PLLA) is great of interest because it is not only a compostable polymer that can be degraded under industrial composting [16] but also exhibits piezoelectric and triboelectric property which transforms mechanical energy to electric polarization, leading to increase PM capturing, up to 99.3% [13,17,18]. PLLA is also considered a tribopositive material, which could generate accumulated charge on the PLLA fiber under ambient vibration [19]. This would increase the electrostatic interaction between PLLA and PM. Moreover, PLLA has been safely used in clinical applications for over 30 years and was approved by FDA in 2004 [20,21]. 

However, air pollution is not only PM but also VOCs, which can cause harmful effects on the public. Thus, a nanofibrous air filter should have the ability to capture PM and entrap VOCs. For this purpose, cyclodextrins (CD) are promising materials due to their unique conical structure. CD are cyclic oligosaccharides consisting of six (α-CD), seven (β-CD), or eight (γ-CD) glucopyranose units linked by α-1,4-glucosidic bonds, which the β-form is the most widely used among them [22]. The typical cavity structure of β-CD with a relatively hydrophobic central cavity and a hydrophilic outer surface allows different guest molecules to form a complex with them. Therefore, applications of β-CD were varied in different fields, such as drug and cosmetic delivery systems [22,23], food packaging [24], and environmental treatment [25,26]. Celebioglu et al. demonstrated that electrospun CD nanofibers can entrap higher amounts of VOCs, including aniline and benzene vapors, than powder form [26]. 

Therefore, this study aimed to develop the electrospun nanofibers from PLLA and CD as an air filter to improve PM and VOC removal efficiency. The obtained electrospun nanofibers, PLLA and CD/PLLA were characterized by their chemical, surface morphology, crystallinity, wettability, and thermal and mechanical properties. Their PM and VOC filtration efficiencies were examined for air filter application. In addition, the triboelectric outputs of PLLA and CD/PLLA nanofibers were also investigated. 

## 2. Materials and Methods

The poly(L-lactic acid) (PLLA) (melt flow rate 6) was supplied by Goodfellow. Methyl-β-cyclodextrin (Mn ~ 1310) was purchased from Sigma Aldrich. Dichloromethane (DCM) and *N,N*-dimethylformamide (DMF) were purchased from RCI Labscan.

### 2.1. Electrospinning of PLLA Filters

PLLA (10% *w*/*v*) solution was prepared by dissolving 10 g of PLLA in 100 mL of mixed solvents, which were DCM and DMF (7/5 *v*/*v*). Then, the solution was sonicated by an ultrasonic bath (GT SONIC-D6 ultrasonic cleaner, 150 W) for 60 min and subsequently stirred at r.t. overnight. After degassing for 60 min, the solution was placed in a 5 mL plastic syringe. The stainless-steel needle (22 G) with a needle length of 30 mm was used to inject the PLLA solution. Cyclodextrin (CD) at concentrations of 2.5, 5, and 10% wt, with respect to PLLA, were mixed into PLLA solution to prepare CD/PLLA nanofiber. The distance between the syringe tip and the aluminum sheet collector distance was 20 cm. The constant feed rate of 0.5 mL/h with 15 kV was applied with an electrospinning system. The obtained electrospun PLLA fibers were dried in a vacuum oven at 60 °C and 10 mbar overnight to remove the residue solvents.

### 2.2. Characterization

The morphology of electrospun PLLA mats was characterized using a scanning electron microscope (SEM) (FEI-NOVA NanoSEM 450). The chemical functionalities of the electrospun fiber were determined by using Fourier transform infrared (FTIR) (Thermo Scientific Nicolet 6700 spectrometer). X-ray diffraction (XRD) patterns were performed on a Bruker D8 ADVANCE diffractometer using CuKα radiation. The chemical state information of the CD/PLLA was determined using XPS (Kratos, Axis Ultra DLD). The nitrogen adsorption isotherms analysis was performed using a gas adsorption instrument (Micromeritics ASAP 2020). Contact angle measurements were determined using a static optical contact angle meter (KINO SL150E). Differential scanning calorimetry (DSC) measurements were performed on a NETZSCH DSC 204 F1 Phoenix with a heat rate of 10 °C/min from 0 °C to 180 °C. Mechanical properties of products were conducted using universal testing machined (Instron 55R4502). The voltage and current outputs of the PLLA-based TENGs were recorded on an oscilloscope (RIGOL DS21202A) and a source meter (Keithley 2400), respectively.

### 2.3. PM Removal Efficiency

The PM_2.5_ removal efficiency of PLLA air filters was tested in a custom-built system, as reported in our previous work [27]. The PLLA air filter with an active area of 7 cm^2^ was placed into a filter bracket. Buddhist incense was used to generate PM particles (at a concentration of ~2000 μg/cm^3^), which was burned in one side of a sealed-testing box. The initial concentration of PM was monitored by a 5 V_DC_ USB PM_2.5_/PM_10_ detector (Air quality dust sensor 2.8″ TFT); 12 V_DC_ fans were used to disperse PM in an air medium inside the testing box. The experiments were tested at r.t. with 80% relative humidity. The amount of PM_2.5_ and PM_10_ were dynamically monitored by an air quality dust sensor for 20 min, recording real-time data every 30 s. The pressure drop (∆P) was measured by a differential pressure meter (Testo 510 pressure manometer). The PM removal efficiency (η), pressure drop (∆P), and quality factor (QF) were calculated by using Equations (1)–(3):

η = C_1_ − C_2_/C_1_ (1)

∆P = P_2_ − P_1_ (2)

QF = ln(1 − η)/∆P(3)

where C_1_ and C_2_ (μg/cm^3^) are the concentration of PM before and after filtration, and P_1_ and P_2_ (Pa) are the air pressure before and after filtration.

### 2.4. VOC Removal Efficiency

The VOC removal efficiency of filters was performed by using formaldehyde as the targeted VOC. The filter with an exposure area of 5 × 5 cm^2^ was placed in a 27 L VOC testing box. Then formaldehyde at a concentration of 1000 ppm was injected into the box through a rubber septum. The formaldehyde concentration before and after absorption was monitored by Extech formaldehyde meter FM300. The data were recorded in real time every 60 s.

### 2.5. Frabication of PLLA-Based Triboelectric Nanogenerators (TENGs)

Electrospun PLLA and CD/PLLA mats were cut into 4 × 4 cm^2^ pieces. The TENG was fabricated in a face-to-face configuration. PLLA or CD/PLLA was attached to aluminum tape and copper tape electrodes, which served as the bottom layer. Kapton film attached with copper tape served as the top layer. 

## 3. Results and Discussion

### 3.1. Electrospinning of PLLA and CD/PLLA

**Figure 1:**
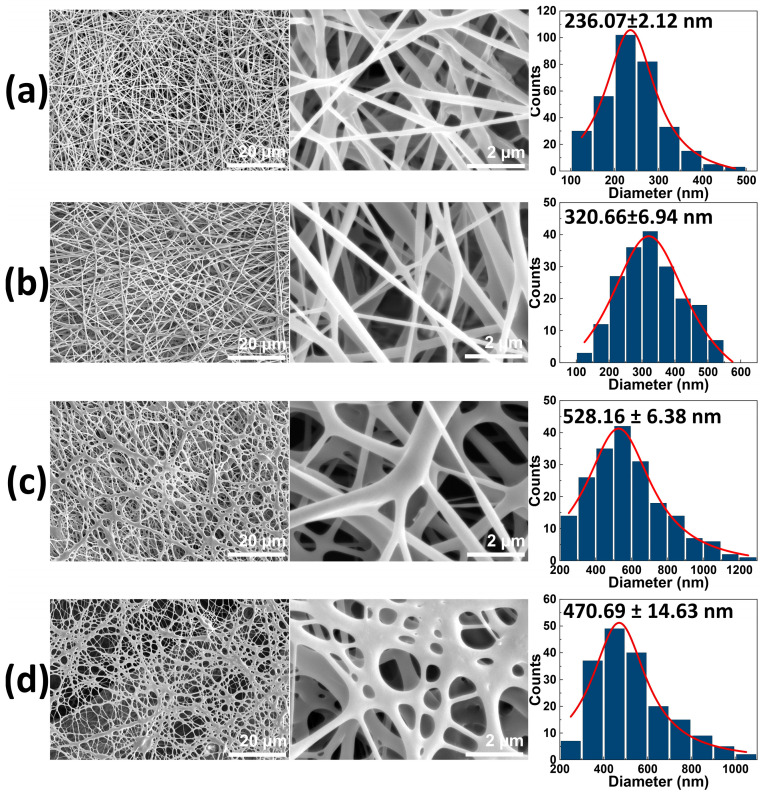
SEM images of (**a**) PLLA nanofiber, (**b**) 2.5 wt% CD/PLLA, (**c**) 5 wt% CD/PLLA, and (**d**) 10 wt% CD/PLLA nanofibers. The third column represents the histograms of size diameter distribution of the corresponding sample.

The PLLA and CD/PLLA nanofibers were successfully fabricated by the electrospinning technique. The morphological structure of electrospun PLLA mats was observed with SEM. As shown in Figure 1a, the non-woven PLLA exhibited continuous and smooth fibers. Its average fiber diameter was in the nanometer range of 236.07 ± 2.12 nm. When CD at concentrations of 2.5, 5, and 10 wt% was mixed with PLLA solution, the morphologies of electrospun CD/PLLA were smooth, as illustrated in Figure 1b,c. However, their fiber diameters were larger than that of the pure PLLA. By increasing CD concentration from 2.5 to 5 wt%, the CD/PLLA fiber diameter was increased from 320.66 ± 6.94 nm to 528.16 ± 6.38 nm. For 10 wt%CD/PLLA, the average fiber diameter was slightly decreased to 470.69 ± 14.63 nm, with less uniformity. The thickness of the electrospun mats was gradually increased from 0.123 ± 0.001 to 0.139 ± 0.005 mm with an increasing CD content. 

Next, the nitrogen adsorption isotherms analysis of electrospun PLLA and CD/PLLA was conducted (Figure S1), and the specific surface area was calculated by the Brunauer-Emmett-Teller (BET) equation (Table 1). PLLA nanofiber possesses a surface area of 20.91 m^2^/g. Incorporation CD with 2.5% wt. can enhance the surface area about 2 folds, as of 40.54 m^2^/g. However, the increasing amount of CD to 5 and 10% wt. resulted in reduced surface area. 

### 3.2. FTIR Spectroscopy

**Figure 2:**
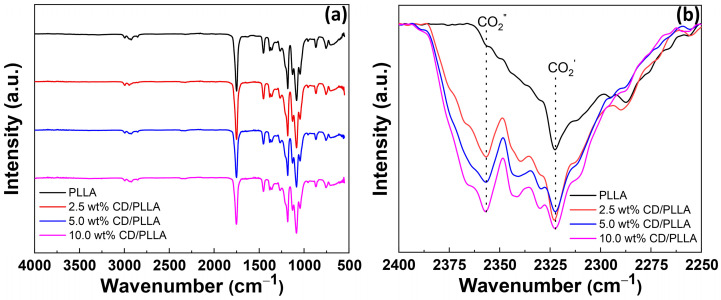
(**a**) FTIR spectra of electrospun PLLA, 2.5 wt % CD/PLLA, 5 wt% CD/PLLA, and 10 wt% CD/PLLA and (**b**) Expanded FTIR spectra.

FTIR was conducted to probe the functionality of electrospun CD/PLLA. FTIR spectrum of PLLA demonstrated peaks at 2995–2926, 2852, 1750, 1452–1365, and 1081 cm^−1^, which corresponded to CH stretching, CH bending, C=O stretching, C-H bending, and C-O stretching, respectively [28]. For CD/PLLA samples, their FTIR spectra were similar to unmodified PLLA because of the similarity of oxygen-containing functional groups. FTIR peak at 2322 cm^−1^ was also observed in all samples, which represented adsorbed CO_2_ on the sample (CO_2_ ′) [29], as shown in Figure 2b. Interestingly, PLLA modified with CD showed a new peak at 2358 cm^−1^. This peak was also assigned to adsorbed gaseous CO_2_ (CO_2_″) but with a different interaction mode [29] compared to CO_2_′ absorbed on the pure PLLA fiber. Moreover, it was found that the intensity of CO_2_′ increased when CD was applied in PLLA, and the intensity of CO_2_″ significantly increased with an increase in CD content. This suggested that CD was embedded in the PLLA fiber, creating a new CO_2_ absorption mode. This result corresponds with the reports that using CD as a green adsorbent could capably adsorb CO_2_ in carbon capture applications [30,31].

### 3.3. X-ray Diffraction (XRD)

**Figure 3:**
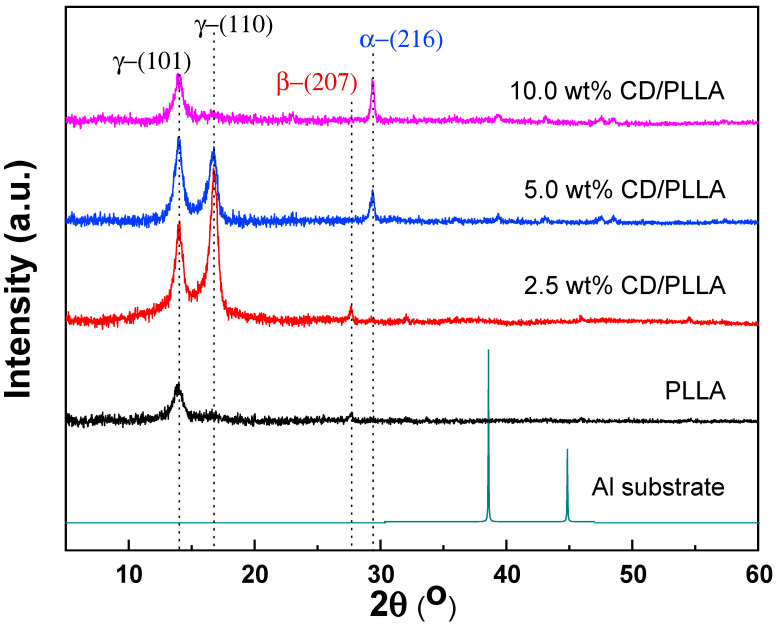
XRD pattern of PLLA and CD/PLLA nanofibers.

The crystal structure of electrospun PLLA nanofibers was investigated by using the X-ray diffraction technique (XRD). As shown in Figure 3, pure PLLA nanofiber showed a diffraction peak at 13.8°, which represents crystal diffraction at the (101) plane of triclinic (ɣ-phase) PLLA (ɣ_(101)_) [32]. This suggests that the orthorhombic gamma phase of PLLA can be formed during the electrospinning process. In addition, a new intense peak located at 16.7°, which refers to the (110) plane of ɣ-phase PLLA (ɣ_(110)_), was evidently observed when 2.5 wt% of CD was mixed in PLLA [32]. Furthermore, it was found that the intensity of ɣ_(110)_ was intensively decreased and disappeared when the content of CD was increased from 5 wt% to 10 wt%; meanwhile, the new peak located at 29.4° emerged, which indicates the (216) diffraction plane of α-phase PLLA [33]. This suggests that CD can induce the crystallization of PLLA from the orthorhombic ɣ-phase to the most stable α-phase of PLLA. Interestingly, the β-phase PLLA located at 27.7° (207) was found in pure PLLA and 2.5 wt% CD/PLLA [33,34]. This existence of β form crystal conformation of PLLA can contribute to great thermal stability, which corresponds well with DSC results and high piezoelectric or triboelectric properties of a material that can be used to support the PM testing results [35]. Thus, it can be concluded that the prepared electrospun fibers composed of different crystal phase structures of PLLA, including PLLA (ɣ_(101)_), 2.5 wt% CD/PLLA (ɣ_(101)_, ɣ_(110)_, β_(207)_), 5.0 wt% CD/PLLA (ɣ_(101)_, ɣ_(110)_, α_(216)_), and 10.0 wt% CD/PLLA (ɣ_(101)_, α_(216)_). From the literature, change in the crystal phase of PLLA could come from various reasons. It was reported that electrospinning of PLLA can result in the metastable β-crystallinity phase [32]. Additionally, cyclodextrin can act as a nucleating agent by making an inclusion complex with PLLA that leads to an enhanced crystallization rate of PLLA [36,37]. As seen in the XRD results, incorporating CD can increase the crystallinity of CD/PLLA nanofiber. In the case of 5 and 10% wt CD/PLLA, the data imply that increasing the content of CD may promote the penetration of PLLA chains into the CD that allows rearrangement of PLLA molecule to form more stable and lower energy α-phase.

### 3.4. X-ray Photoelectron Spectroscopy (XPS)

**Figure 4:**
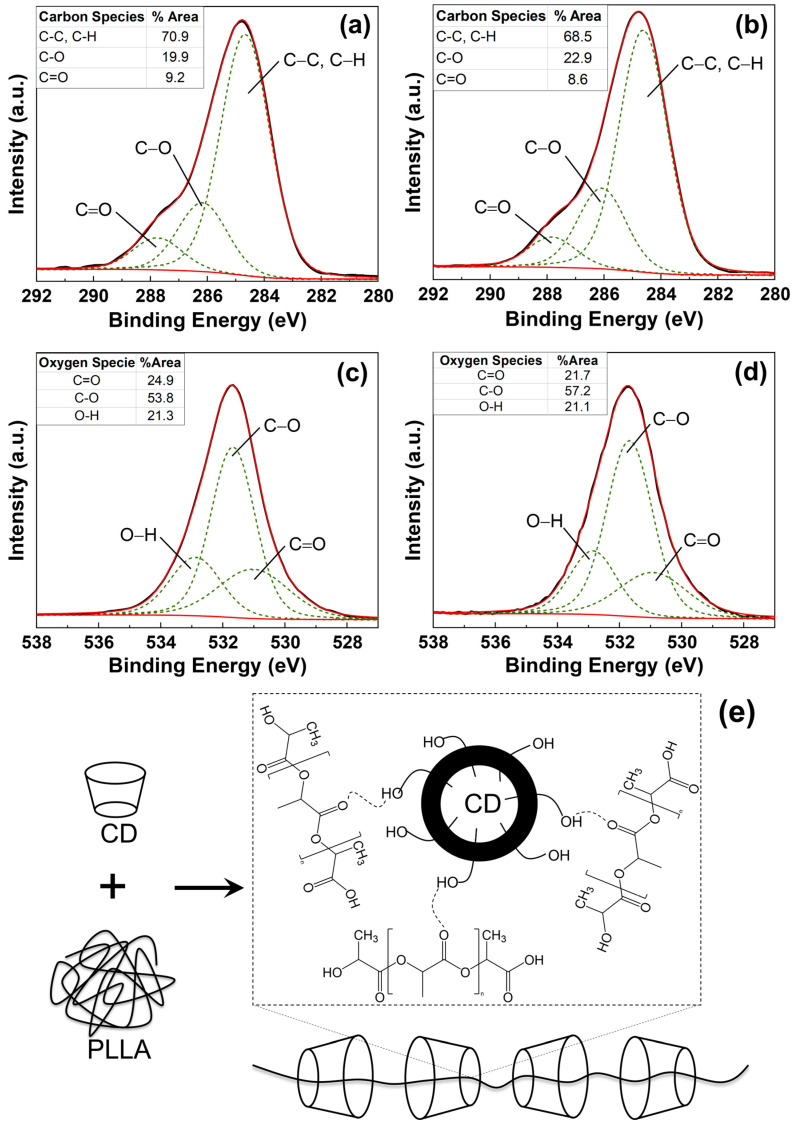
C1s XPS spectra of (**a**) PLLA and (**b**) 2.5 wt% CD/PLLA and O 1s XPS spectra of (**c**) PLLA, (**d**) 2.5 wt% CD/PLLA electrospun nanofibers. (**e**) the schematic illustration of the interaction between CD and PLLA.

The chemical state of CD blended with PLLA was investigated by X-ray photoelectron microscopy technique. The O1s and C1s of the XPS spectrum of pure PLLA and 2.5 wt% CD/PLLA were deconvoluted by using CasaXPS software coupled with Shirley as the baseline. As shown in Figure 4a, the XPS C 1s spectrum of pure PLLA electrospun fiber can be fitted into three peaks centered at 284.7 eV, 286.2 eV, and 287.6 eV, which could be indexed to the sp^3^ hybridized carbon (C-C) and C-H, C-O-C, and C=O bonds, respectively [38]. For 2.5 wt% CD/PLLA (Figure 4b), the C1s XPS spectrum was also deconvoluted into three peaks, including 284.5 eV (C-C, C-H), 285.6 (C-O-C + C-OH), and 287.5 (C=O). In addition, it was found that the C-O peak of 2.5 wt% CD/PLLA was significantly shifted to the lower binding energy. This is because the additional C-OH and C-O-C originated by CD in PLLA. The percent content of each carbon species of pure PLLA and 2.5 wt% CD/PLLA was summarized in Figure 4 (inset). It was shown that the percentage of C-O species was significantly increased when CD was applied to PLLA because of C-OH and C-O-C addition. These oxygen-containing functional groups can promote a hydrophilic exterior of the filter, which may affect the filtration efficiency and the degradation of volatile organic compounds’ performance.

The interaction between PLLA and CD was elucidated by XPS O 1s spectra, as shown in Figure 4c,d. The XPS O 1s spectrum of pure PLLA electrospun fiber can be fitted into three peaks centered at 531.0, 531.7, and 532.9 eV, which could be indexed to C=O, C-O, and O-H, respectively [39,40]. It was found that the percentage of C-O was significantly increased when CD was integrated into PLLA. This corresponds well with XPS C 1s results, as shown in Figure 4b. Interestingly, the deconvoluted peak at C=O of 2.5 wt% CD/PLLA was red-shifted (from 531.1 to 530.7 eV). This finding implies that the lower electronegativity element interacted with oxygen in C=O bonds. As a result, the electron density around the oxygen atom increases, and the binding energy decreases. Thus, the possible interaction between CD and PLLA is the hydrogen bonding between the carbonyl group of PLLA and the hydroxyl group of CD (C=O- - -H-O), as illustrated in Figure 4e.

### 3.5. Water Contact Angle Measurement

**Figure 5:**
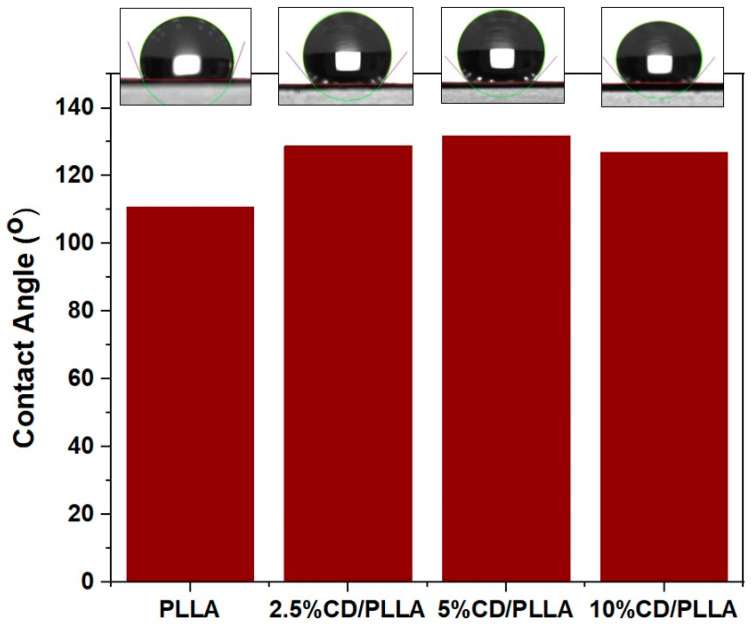
Contact angle of electrospun PLLA compared with 2.5, 5, and 10 wt% CD/PLLA.

The hydrophobicity characteristic of PLLA and CD/PLLA nanofibers was investigated using contact angle measurements of pure water. As shown in Figure 5. The contact angle of electrospun PLLA was 110.6°, indicating that the surface of PLLA nanofiber was hydrophobic. The contact angles of electrospun 2.5, 5, and 10 wt%CD/PLLA were 128.8°, 131.8° and 126.9°, respectively. The increased water contact angle of CD/PLLA suggests that mixing CD into PLLA could increase their hydrophobicity. This non-wetting characteristic could benefit using CD/PLLA nanofiber as an air filter because the air filter should have water-repellent properties to protect it from liquid aerosol and condensed droplets.

### 3.6. Differential Scanning Calorimetry (DSC)

**Figure 6:**
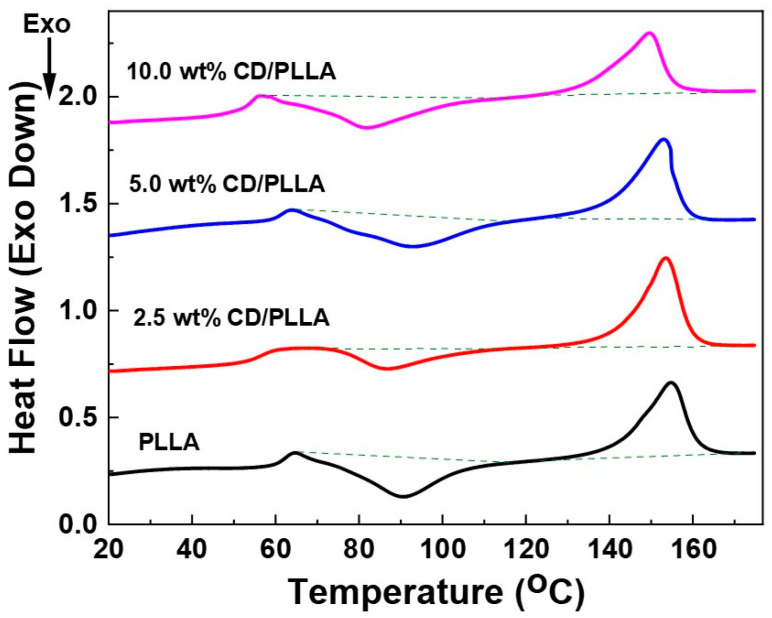
DSC thermograms of PLLA and CD/PLLA nanofibers.

Thermal properties of electrospun PLLA and CD/PLLA were assessed by using DSC. Figure 6 shows DSC thermograms of electrospun PLLA and CD/PLLA. The thermal properties are summarized in Table 2. Neat PLLA nanofiber, a semi-crystalline polymer, exhibited glass transition temperature (T_g_) at 59.6 °C. This result was similar to the previous studies [41,42]. It was found that incorporating a small amount of CD with PLLA resulted in decreasing T_g_. The decrease in T_g_ of PLLA blended with CD was also previously found in the literature [43,44]. It was explained that CD/PLLA can trap residual solvents in the polymer matrix, which behave as a plasticizer and reduce the T_g_ [43,45]. The melting temperature (T_m_) of PLLA nanofiber was 141.5 °C. The T_m_ of CD/PLLA tended to decrease with a higher concentration of CD, indicating a variation in the degree of crystallinity of CD/PLLA nanofibers. 

### 3.7. Tensile Strength

**Figure 7:**
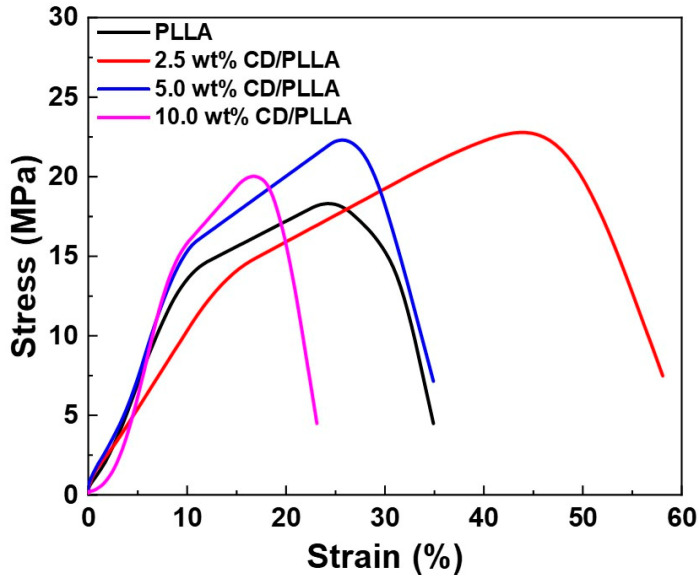
Stress-strain curves of PLLA and CD/PLLA nanofibers.

The mechanical properties of PLLA and CD/PLLA nanofibers were characterized by tensile measurement. Figure 7 illustrates the stress-strain curved of PLLA and CD/PLLA nanofibers. The mechanical parameters, including tensile strength, Young’s modulus, and elongation at break, are summarized in Table 3. Embedding CD with PLLA provided a higher tensile strength of CD/PLLA nanofibers. The highest tensile strength of 22.78 ± 1.53 MPa was obtained from 2.5 wt% CD/PLLA. The tensile strength was slightly decreased with an increasing amount of CD. It should be noted that the elongation at break of 2.5 wt% CD/PLLA is significantly increased. This high elasticity could be due to the lowest T_g_ of 2.5 wt% CD/PLLA and solvent retention that may affect the nanofibers to be more elastic [45]. 

### 3.8. PMs Removal Efficiency

**Figure 8:**
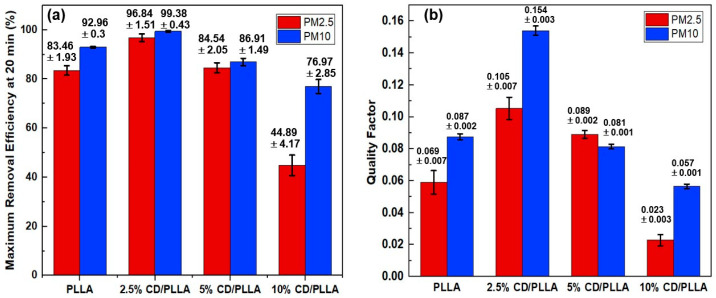
(**a**) PM_2.5_ and PM_10_ removal efficiency and (**b**) quality factor of PLLA and CD/PLLA nanofibers.

PM_2.5_ and PM_10_ removal efficiencies were measured in a sealed-custom testing box which was modified from our previous work [27]. As shown in Figure 8a, the highest removal efficiency of PM_2.5_ (96.84 ± 1.51%) and PM_10_ (99.38 ± 0.43%) was achieved with 2.5 wt% CD/PLLA nanofibers, followed by PLLA, 5 wt% CD/PLLA, and 10 wt% CD/PLLA, respectively. Since the thickness of each nanofiber was in the same range (1.2–1.4 mm), the difference in PM removal performance can be described by the surface area and void area of each nanofiber (Table 1 and Figure S2). For 2.5 wt% CD/PLLA, it has the highest surface area of 40.54 m^2^/g and the highest porosity (Figure S2b,e), implying the efficient filter PM_2.5_ and PM_10_. In the case of 10 wt% CD/PLLA, its surface area was significantly low (1.23 m^2^/g), and the void area was obviously larger than the other samples (Figure S3d), resulting in the PM_2.5_ passing through easily, and caused the lowest PM removal efficiency. 

Quality factor (QF) is generally indicated the quality of the filter. The best air filter, 2.5 wt% CD/PLLA, showed a high QF of 0.105 for PM_2.5_ filtration. The QF value is better than the similar work by Zhang et al. (QF = 0.047) [13] because our CD/PLLA has a lower pressure drop as of 42.87 ± 2.79. 

### 3.9. VOC Removal Efficiency

**Figure 9:**
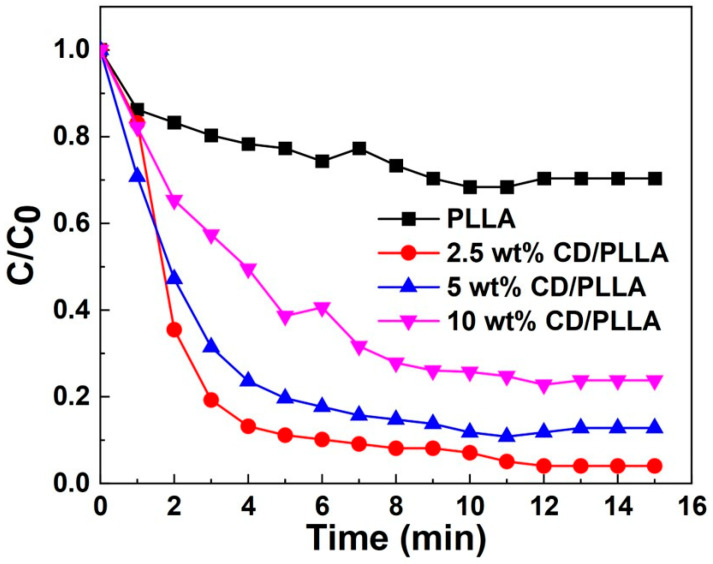
VOC removal efficiency of PLLA and CD/PLLA nanofibers.

VOC entrapment capability was investigated by using toluene vapor. As evidenced by Figure 9, CD/PLLA nanofibers exhibited higher entrapment of toluene amount than the plain PLLA nanofiber. This result could be explained by the formation of an inclusion complex of toluene in CD. The 2.5 wt% CD/PLLA showed the highest VOC removal efficiency up to 90% within 5 min, followed by 5 wt% CD/PLLA and 10 wt% CD/PLLA. It was found that a higher amount of CD resulted in a lower VOC entrapment. This suggested that the embedded CD moiety in PLLA nanofibers was not equally exposed and accessible to trap toluene.

### 3.10. Triboelectric Performance

**Figure 10:**
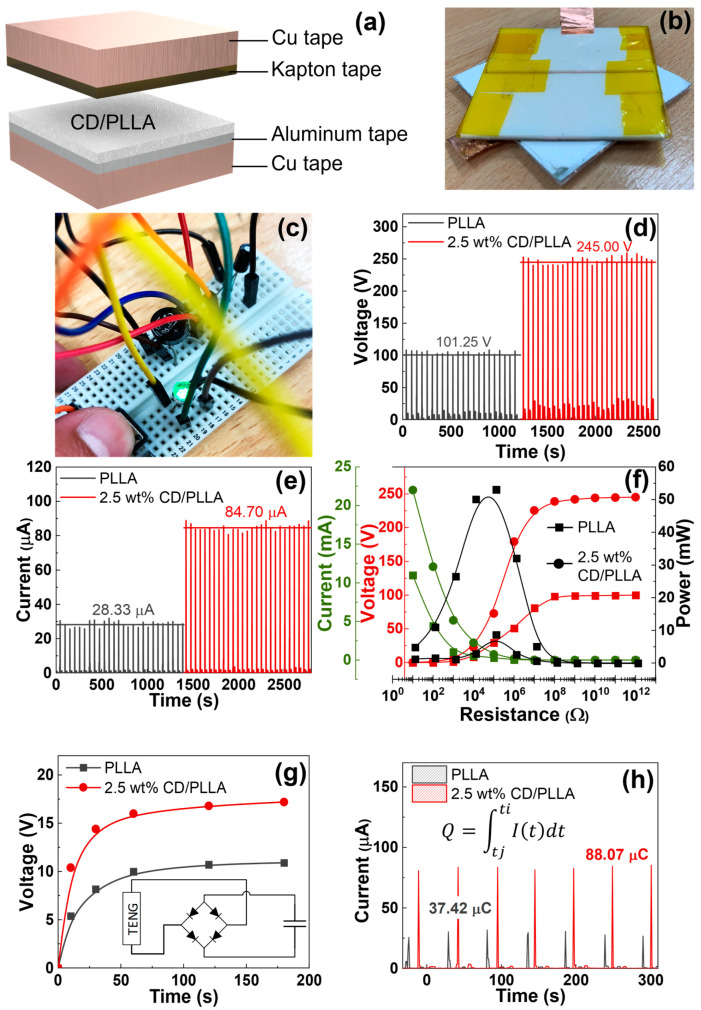
(**a**) Configuration and (**b**) photograph of CD/PLLA nanofiber-based TENG, (**c**) photograph of CD/PLLA nanofiber-based TENG can a charge capacitor to light, and LED, (**d**) output voltage and (**e**) output voltage of PLLA and 2.5 wt% CD/PLLA nanofibers based TENG, (**f**) output voltage/current and output power density when connected with external load resistance, (**g**) charging curves of a capacitor (2.2 μF) and the schematic illustration of charging circuit (inset), and (**h**) the average calculated surface charge.

To investigate the triboelectric output of PLLA and CD/PLLA nanofibers, the nanofibers were utilized to fabricate triboelectric nanogenerator (TENG) devices in a face-to-face configuration, as shown in Figure 10a,b. According to the triboelectric series [19], PLLA is a positive triboelectric material in comparison to Kapton film. The electrospun PLLA nanofiber layer served as the bottom positive layer, and the Kapton film taped with copper tape aluminum served as the top negative layer. Figure 10d,e show the corresponding electrical outputs of PLLA-TENG and 2.5 wt% CD/PLLA-TENG under periodic tapping (force ~2N). The plain PLLA-TENG exhibited an average output voltage of 101.25 V and an average output current of 28.33 μA. The electrical outputs of 2.5 wt% CD/PLLA-TENG were significantly increased to 245 V and 84.70 μA, resulting in an output current density of 5.29 μA/cm^2^. In comparison with our previous work on TENG [46], this electrical performance was sufficient to directly light more than 100 LEDs. For the 2.5 wt% CD/PLLA-TENG, the average calculated surface charge under pressing was 88.07 μC (Figure 10h). The enhancement of electrical outputs of 2.5 wt% CD/PLLA-TENG could be contributed by hydrophobicity, crystallinity, and surface roughness of the material. From the contact angle measurement (Figure 5), it showed that the surface of 2.5 wt% CD/PLLA nanofiber is more hydrophobic, which can increase charge separation and charge accumulation [46,47]. Together with the XRD result, 2.5 wt% CD/PLLA nanofiber has higher crystallinity than that of pure PLLA. Thus, 2.5 wt% CD/PLLA is tended to be a more tribopositive material [48]. Additionally, mixing CD with PLLA could increase the surface roughness of the nanofiber, which was evidenced by BET results (Figure S1), showing that 2.5 wt% CD/PLLA has the highest surface area that could consequently improve the friction area of the TENG. Figure 10f shows the dependence output voltage and power density of PLLA and 2.5 wt% CD/PLLA on load resistance (from 10 Ω–1000 GΩ.). It was shown that the voltage linearly increased, followed by the voltage saturation at a load resistance of 1 GΩ. As a result, the power density of 2.5 wt% CD/PLLA TENG provided a maximum value of 53.04 mW/cm^2^ at an external resistance of 0.1 MΩ. In addition, the charging behaviors of PLLA-TENG and 2.5 wt% CD/PLLA-TENG were determined using a capacitor (2.2 μF). As depicted in Figure 10g, 2.5 wt% CD/PLLA-TENG can charge a capacitor to reach a voltage of 15 V within 60 s, while PLLA-TENG can charge a capacitor to reach 10 V within 60 s. Figure 10c demonstrates that a charged capacitor was used as an external storage to light an LED. These remarkable triboelectric outputs of CD/PLLA could give a benefit of PM capturing by increasing the accumulated triboelectric charge at the nanofiber surface under ambient vibrations such as particle collision and airflow.

## 4. Conclusions

In conclusion, biodegradable PLLA, 2.5 wt% CD/PLLA, 5 wt% CD/PLLA, and 10 wt% CD/PLLA nanofibers were successfully produced as smooth and continuous nanofibers. The 2.5 wt% CD/PLLA, which have the smallest fiber diameter, the highest surface area, and porosity, showed the best properties for air pollutions removal, including PM_2.5_ (96.84 ± 1.51%), PM_10_ (96.84 ± 1.51%), and VOC filtration. In addition, 2.5 wt% CD/PLLA can provide better triboelectric outputs, with maximum output voltage of 245 V and output current of 84.70 μA. The triboelectric property would be useful to capture PM via electrostatic interaction. These demonstrated that 2.5 wt% CD/PLLA is suitable to utilize as environmental-friendly air filter. 

## Figures and Tables

**Table 1:** Fiber diameter, thickness, and surface area of PLLA and CD/PLLA nanofibers.

Samples	Fiber Diameter (nm)	Thickness (mm)	Surface Area (m^2^/g)
PLLA	236.07 ± 2.12	0.123 ± 0.001	20.91
2.5 wt% CD/PLLA	320.66 ± 6.94	0.125 ± 0.001	40.54
5 wt% CD/PLLA	528.16 ± 6.38	0.132 ± 0.003	11.27
10 wt% CD/PLLA	470.69 ± 14.63	0.139 ± 0.005	1.23

**Table 2:** Summary of T_g_, enthalpy at T_g_, Tm, and enthalpy at T_m_ of PLLA and CD/PLLA nanofibers.

Samples	T_g_ (°C)	Enthalpy at T_g_ (J/g)	T_m_ (°C)	Enthalpy at T_m_ (J/g)
PLLA	59.6	−25.77	141.5	26.21
2.5 wt% CD/PLLA	51.6	−11.77	142.8	28.72
5 wt% CD/PLLA	59.0	−25.19	141.0	25.53
10 wt% CD/PLLA	50.9	−22.87	134.6	21.34

**Table 3:** Summary of tensile strength, Young’s modulus, and elongation at break of PLLA and CD/PLLA nanofibers.

Samples	Tensile Strength (Mpa)	Young’s Modulus (MPa)	Elongation at Break (%)
PLLA	18.31 ± 1.01	1.70 ± 0.18	34.89 ± 3.54
2.5 wt% CD/PLLA	22.78 ± 1.53	0.98 ± 1.11	54.09 ± 2.34
5 wt% CD/PLLA	22.30 ± 1.82	1.82 ± 0.19	34.78 ± 2.20
10 wt% CD/PLLA	20.02 ± 1.00	2.21 ± 0.34	23.12 ± 4.20

## Data Availability

Not applicable.

## Supplementary Materials

The following supporting information can be downloaded at: https://www.mdpi.com/article/10.3390/polym15030722/s1, Figure S1: BET adsorption isotherm of PLLA and CD/PLLA nanofibers; Figure S2: Voids (in red color) of (a) PLLA, (b) 2.5 wt% CD/PLLA, (c) 5 wt% CD/PLLA, (d) 10 wt% CD/PLLA nanofibers, and (d) void density plot.
