# Supplementary material for: Electrospun Cyclodextrin/Poly(L-lactic acid) Nanofibers for Efficient Air Filter: Their PM and VOC Removal Efficiency and Triboelectric Outputs
Source: Polymers (Basel). 2023 Jan 31;15(3):722. doi: 10.3390/polym15030722 (PMC9921114; doi:10.3390/polym15030722)
Supplement: Supplementary file 1 [file polymers-15-00722-s001.zip › polymers-2181952-supplementary.pdf]

Supplementary Information

Sompit Wanwong [1,*], Weradesh Sangkhun [1] and Pimsumon Jiamboonsri [2]

1 Materials Technology Program, School of Energy, Environment and Materials, King Mongkut's University of Technology Thonburi, 126 Pracha Uthit Road, Bang Mod, Thung Khru, Bangkok 10140, Thailand
2 Faculty of Medicine, King Mongkut's Institute of Technology, Bangkok 10520, Thailand

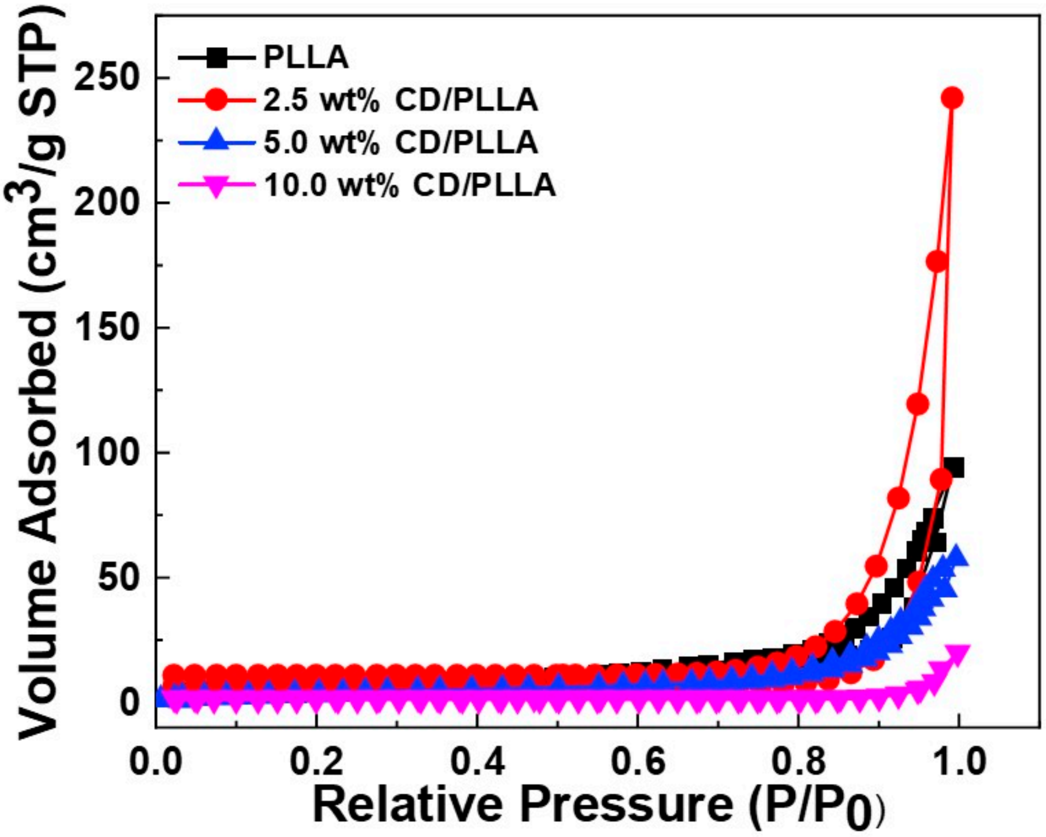

Figure S1. BET adsorption isotherm of PLLA and CD/PLLA nanofibers.

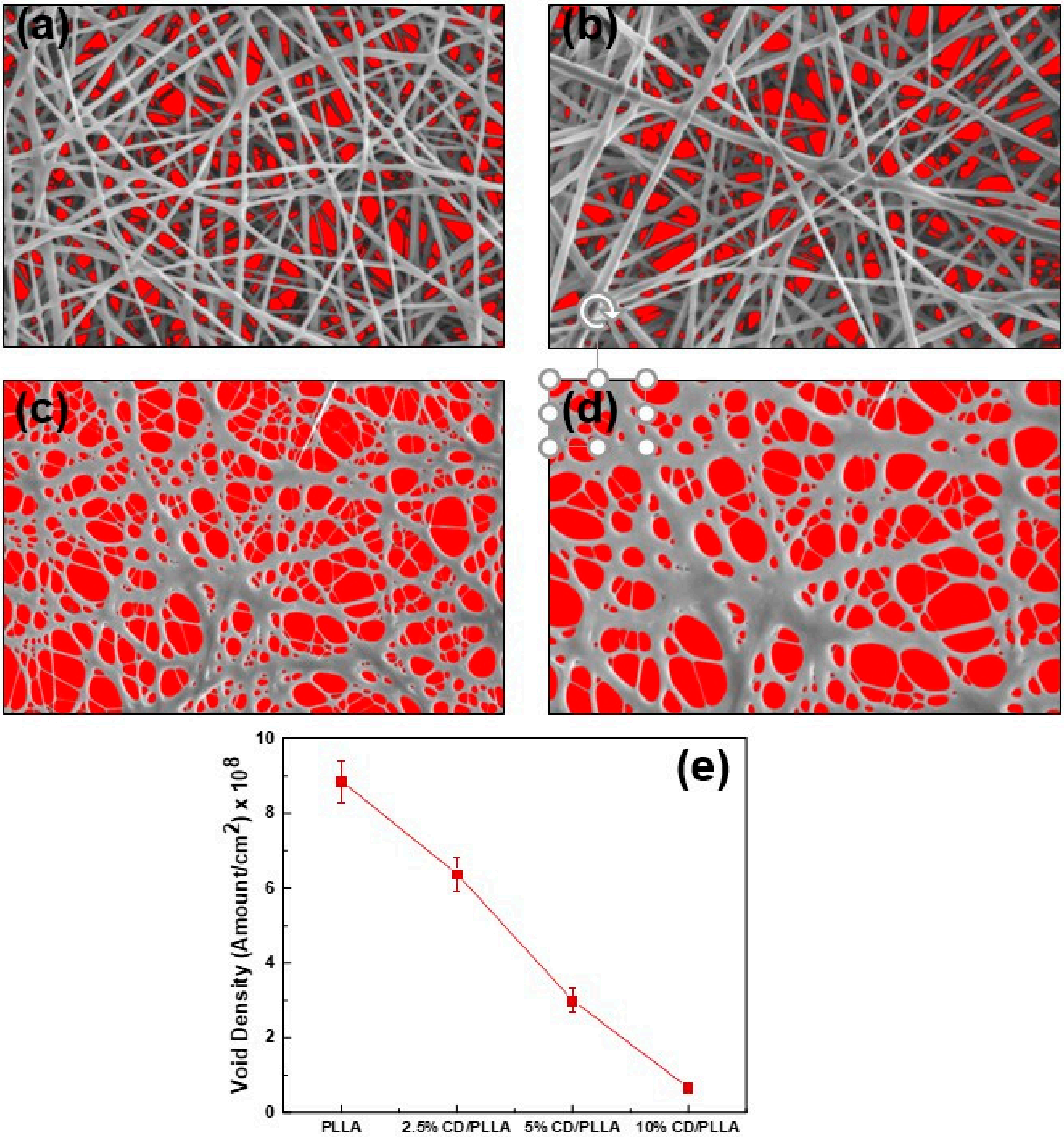

Figure S2. Voids (in red color) of (a) PLLA, (b) 2.5wt% CD/PLLA, (c) 5wt% CD/PLLA, (d) 10wt% CD/PLLA nanofibers and (e) void density plot.

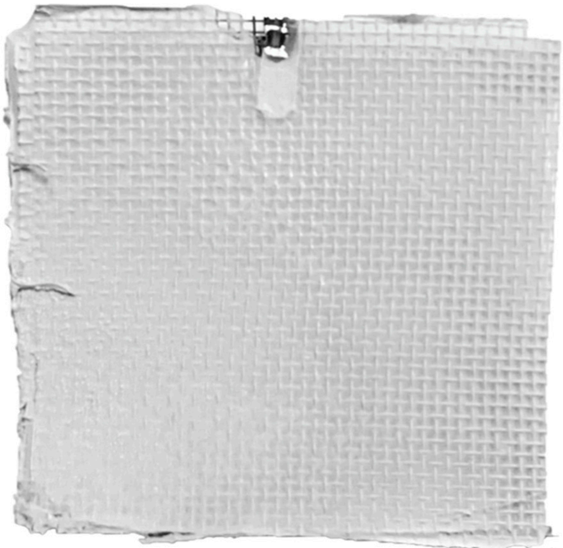

Figure S3. Photograph of electrospun CD/PLLA mat
